# Patients exposed to vancomycin-resistant enterococci during in-hospital outbreaks in a low endemic setting: a proposal for risk-based screening

**DOI:** 10.1186/s13756-022-01089-9

**Published:** 2022-04-13

**Authors:** Andrea C. Büchler, Silvio Ragozzino, Melanie Wicki, Violeta Spaniol, Sammy Jäger, Helena M. B. Seth-Smith, Daniel Goldenberger, Vladimira Hinic, Adrian Egli, Reno Frei, Andreas F. Widmer

**Affiliations:** 1 Division of Infectious Diseases and Hospital Epidemiology, University Hospital Basel, Petersgraben 4, 4031 Basel, Switzerland; 2 Clinical Bacteriology and Mycology, University Hospital Basel, University of Basel, Basel, Switzerland; 3 Applied Microbiology Research Laboratory, University of Basel, Basel, Switzerland

**Keywords:** Vancomycin-resistant enterococci, Screening, Outbreak, Contact investigations, Infection control

## Abstract

**Background:**

The optimal extent of screening of contact patients (CoPat) after exposure to patients infected or colonized with vancomycin-resistant enterococci (VRE) remains controversial.

**Methods:**

We retrospectively developed a new risk stratification for screening patients exposed to VRE, based on data from three outbreaks—two with *Enterococcus faecium vanB* and one with *Enterococcus faecium vanA* involving 1096 CoPat—in a low endemic setting. We classified them into four risk groups: three on environmental exposure, one by healthcare exposure: high (sharing the same room/bathroom with a VRE-colonized patient), medium (hospitalization in the same room after a VRE-colonized patient’s discharge until terminal disinfection including ultraviolet C (UVc)-disinfection), low (hospitalized in the same room within three weeks before the VRE-colonized patient), and “staff” (screening of patients having the same medical care team).

**Results:**

VRE-transmission occurred in 7.9% in the high-risk group compared to 0.6% and 0% in the medium and low risk groups. There was a significant trend to higher rates of transmission by risk level of exposure (p < 0.001). In the “staff” group, VRE transmission rate was 2.3%.

**Conclusion:**

Based on this stratification, we recommend to focus screening of exposed CoPat on the high-risk and “staff” group, saving resources and costs, but larger studies will allow to further improve the yield of VRE screening in the outbreak setting.

## Background

Outbreaks of vancomycin-resistant enterococci (VRE) are an emerging problem of multidrug-resistant microorganisms (MDRO) even in low endemic countries such as Switzerland [1]. The CDC’s Containment Strategy includes rapid identification, infection control assessments, colonization screenings when needed, coordinated response between facilities and continuation of assessments and colonization screenings until the spread is controlled, without specifying the recommended measures, in particular the patients needed to screen [2]. The Swiss national guidelines recommend contact precautions for patients colonized or infected with VRE. Screening is recommended for any patient potentially exposed to the index patient during the preceding four weeks. In addition, patients exposed to VRE-infected or colonized patients should also be kept under contact precautions as long as screening results are pending [3]. The guideline was issued in 2018 after one of the largest VRE outbreak in Switzerland was registered with more than 500 colonized or infected patients involved [4]. This epidemic has challenged the healthcare system in Switzerland, given the fact that many patients are transferred between healthcare institutions [5], and many hospitals reported clusters traceable to the source hospital. The evidence for contact precautions is based on cohort studies during outbreaks, but no strong clinical trial supports its use in the endemic setting [6]. The effect of routine glove and gown use for patients in intensive care units (ICUs) failed to demonstrate a significant effect on VRE-transmission [7]. In addition, VRE-colonized patients rarely proceed to infections, except in immunocompromised patients who are at increased risk of developing bloodstream infections [8]. Therefore, some institutions decided to discontinue contact precautions for VRE-colonized patients without recognized increase in transmission or infection rates [9, 10]. In addition, noninfectious hospital adverse events, such as falls, declined after lifting contact precautions [11]. After experiencing three outbreaks with VRE at our institution with 1096 contact patients (CoPat) involved, the impact of preemptive contact precautions for patients exposed to VRE-colonized or -infected patients was challenged to be a reasonable approach to limit the spread of VRE in the outbreak setting: the lack of isolation rooms, the additional costs for contact precautions and the additional workload for healthcare workers triggered a change of our policy [12]. The group of Marc Bonten suggested preemptive isolation of high-risk patients as part of the successful control of VRE outbreaks, but the term high-risk was not well defined [13]. Therefore, we retrospectively stratified CoPat by level of exposure to a VRE-colonized or -infected patient, and evaluated the rate of transmission during three outbreaks with *Enterococcus faecium vanB* and *vanA* in a low endemic setting.

## Methods

The University Hospital Basel, Switzerland, is an 850-bed tertiary care center with a large stem cell and renal transplantation center and approximately 38,000 admissions per year. Routine surveillance of MDRO includes screening of patients repatriated from abroad for methicillin-resistant *Staphylococcus aureus* (MRSA), VRE, extended-spectrum betalactamase-producing Gram-negatives (ESBL) and carbapenemase-producing Gram-negatives (CPGN). Daily cleaning is extended to terminal disinfection of rooms after patient discharge with a surface disinfectant, a combination of quaternary ammonium compounds and aldehydes (Deconex®, Borer Chemie AG, Switzerland). However, when a patient with VRE or CPGN has occupied a room, ultraviolet C (UVc) light device (UVDI-360 room sanitizer, Valencia, California, USA) application twice for 20 min routinely followed terminal disinfection [14].

### Outbreak description

**Fig. 1:**
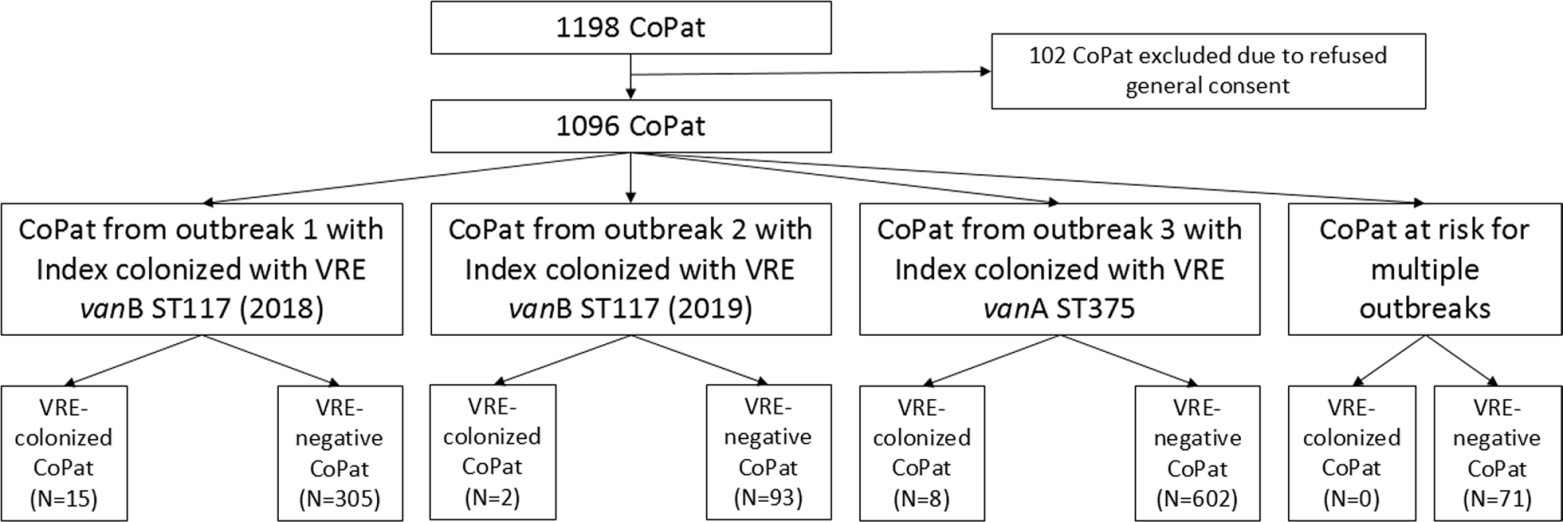
Flowchart of contact patients (CoPat) of the three outbreaks from 2018–2019. VRE = vancomycin-resistant enterococci

Between 2018 and 2019, we experienced two outbreaks with VRE *vanB* (patients n = 16 and n = 3, respectively) and one outbreak with VRE *vanA* (patients n = 9) from three index patients, all of them *E. faecium*. An outbreak was defined as one newly positive tested patient. For the analyses, we excluded all CoPat and index patients with a refused general consent, resulting in the analysis of two index patients (both VRE *vanB*) and 1096 patients at risk for VRE *vanB* and/or VRE *vanA* (Fig. 1). Cases were defined as patients infected or colonized (defined as VRE detection without any clinical signs of infection) with VRE during the outbreak period with an epidemiological link and a related strain to the index patient’s strain confirmed by whole genome sequencing (WGS). CoPat were defined as exposed to a VRE-infected or colonized patient with either direct contact (hospitalization in the same room or sharing the same bathroom) or indirect contact through the same environment or staff (hospitalization in the same room within the preceding three weeks or hospitalization in the same room after the index patient until terminal disinfection including UVc disinfection was performed).

The outbreaks were contained after the introduction of several infection control measures, including contact isolation of colonized or infected patients, preemptive contact isolation of risk patients in the high, medium, and low risk groups, cohorting of the CoPat according to their risk status and with designated staff for each cohort, enhanced disinfection of the patient’s room and the shared bathrooms. Infected or colonized patients as well as patients at risk for colonization were tagged in the electronic hospital information system. Ward-wide screening was performed on the affected general medical and surgical wards and screening on admission after transfer from another hospital of the same country was introduced. Monthly screening of hematological patients was already performed before the outbreaks, since they represent a high-risk group for infections with VRE, and was continued unmodified. CoPat were screened weekly during their hospitalization and non-hospitalized CoPat were screened in an ambulatory setting by rectal screenings in the outpatient clinic or by a kit sent to their homes for stool sample collection. CoPat were defined negative after three consecutive culture-negative rectal screenings (at least one week apart) or single negative stool sample at the earliest eight weeks after exposure. Environmental screening of the affected wards including the shared bathrooms was performed.

### Screening and isolation of contact patients

Based on the Swiss national guidelines, we screened all patients after exposure with VRE-infected or colonized patients. Additionally, we screened all patients who had been hospitalized in the same room as the index patient in the preceding three weeks to identify an earlier index patient, as well as patients hospitalized in the same room after discharge of the index patient until terminal disinfection including UVc light disinfection. This situation only occurred when patients were screened positive for VRE after discharge at our institution. We classified the CoPat into four risk groups:

1. High: defined as sharing the same room and/or bathroom with a VRE-colonized patient.
2. Medium: defined as hospitalization in the same room after a VRE-colonized patient’s discharge until terminal disinfection including UVc light disinfection.
3. Low: defined as hospitalized in a room within three weeks before the VRE-colonized patient.
4. ”Staff”: defined as all patients having the same medical care team as the index or another VRE-positive patient and therefore being screened by ward-wide screenings.

Screening was done during hospitalization or when the patient was readmitted if this was within eight weeks. Patients not hospitalized within eight weeks were screened in an ambulatory setting. Ambulatory screening was performed with either three consecutive rectal swabs (at least one week apart) or one stool sample.

### Microbiology and molecular typing

Any hospitalized VRE-exposed patient was screened using three consecutive rectal swabs (at least one week apart; eSwab™, Copan Group, Brescia, Italia); ambulant patients were screened by either three consecutive rectal swabs (at least one week apart) or one stool sample. Samples were processed in VRE enrichment broth (bioMérieux, Geneva, Switzerland) supplemented with 6 mg/L vancomycin for 24 h followed by ChromID VRE agar incubated for 48 h (bioMérieux, Geneva, Switzerland). We identified the species using matrix-assisted laser desorption/ionization time-of-flight mass spectrometry (MALDI Biotyper, Bruker Daltonics, Bremen, Germany) and susceptibility testing was performed by VITEK 2 (bioMérieux Inc., Durham, North Carolina, USA). Presence of *vanA* or *vanB* was confirmed using the Xpert *vanA/vanB* assay (Cepheid Inc., Sunnyvale, California, USA). All isolates were further characterized by whole genome sequencing (WGS) on MiSeq or NextSeq500 platforms (Illumina, San Diego, California, USA) after library preparation using NexteraXT or Nexteraflex (Illumina) to minimum 40 × mean coverage and were stored at − 80° Celsius as previously described [4]. All read data has been submitted to the European Nucleotide Archive (ENA) under project number PRJEB41046. Multilocus sequence typing (MLST) and core genome MLST (cgMLST) were defined within Ridom Seqsphere + v6.0.2 [15], using the defined scheme [16]. Outbreaks were defined as having no more than 20 alleles between isolates and being epidemiologically related.

### Statistical analysis

The statistical analyses were done using Python 3.8.5 (pandas 1.1.2, numpy 1.19.1, scipy 1.5.0, statsmodels 0.11.1). Continuous variables were expressed as median and interquartile range (IQR) and categorical variables as frequencies and percentages. Categorical variables were analyzed by chi-square test, and Cochran-Armitage test for linear trends as applicable. Continuous variables were analyzed by Wilcoxon Rank-Sum test. P-values of less than 0.05 were considered as significant.

Data were generated as part of the ongoing quality improvement program not requiring approval from the human subject committee: however, patients who refused general consent for using their fully anonymized data for clinical research were excluded. All analysis were performed after removal of any identification of individual patient data.

## Results

**Fig. 2:**
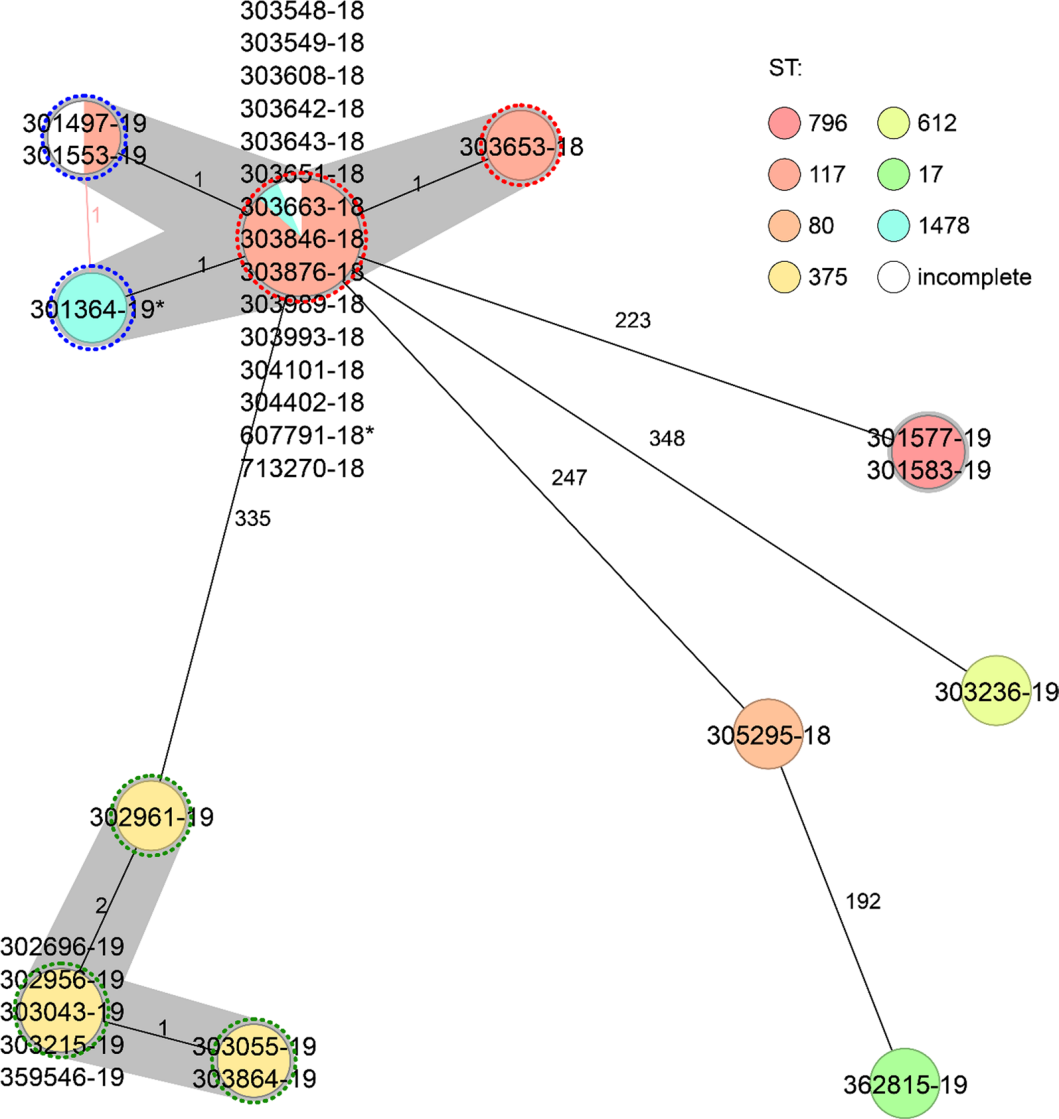
cgMLST minimum spanning tree of the three outbreaks as well as the five non-related strains detected during the same period. The strains belonging to outbreaks are shown with coloured outlines: first, red; second, green; third, blue. The final numbers of isolate names indicate the year of isolation, and index cases are indicated with *. In two cases MLST could not be defined, as single MLST targets were not found in the genomes. In two cases within outbreak one, the MLST was called as ST1478, a single locus variant from ST117 in *pstS*

During 2018 to 2019, we experienced three outbreaks—two with *E. faecium vanB* ST117 and one with *E. faecium vanA* ST375, never detected before in our hospital. The VRE-colonization rate at our institution was extremely low with 0.042 and 0.066/1,000 patient days in 2016 and 2017, respectively, but increased with the outbreaks to 0.216 and 0.155/1,000 patient days in 2018 and 2019, respectively [17]. Overall, 1198 CoPat were exposed to VRE-colonized or –infected patients and we had to exclude 102 due to refused general consent. Therefore, 1096 CoPat were included in the analysis (681 with *vanA* ST375 and 486 with *vanB* ST117) (Fig. 1). Of the CoPat, 53.6% were male and the median age was 70 (IQR 57–80) years (Table 1). The cgMLST minimum spanning tree of all detected VRE strains during the outbreak period confirmed the epidemiological links clustering in three outbreaks (Fig. 2). Only five epidemiologically non-related VRE isolates were identified during the outbreak period, all of them not clustering with the outbreak strains according to the WGS typing. The outbreaks were contained by increased and extensive infection control measures as described in the method section.

**Table 1:** Characteristics of VRE-positive and VRE-negative patients of all three outbreaks

	VRE-positive patients (N = 27)	VRE-negative patients (N = 1067)	*p *value*
Age, median (IQR)	80 (67–86)	70 (56–80)	0.01
Male sex, n (%)	12 (44.4)	579 (54.1)	0.43
VRE-unrelated death during follow-up, n (%)	2 (7.4)	82 (7.7)	0.75

*Chi-square test for categorical variables, Wilcoxon Rank-Sum test for continuous variables

### Risk based screening for CoPat—a proposal

Based on three outbreaks with VRE, we retrospectively developed a risk stratification of CoPat based on their exposure to a VRE-colonized or -infected patient. VRE-transmission occurred in 7.9% in the high-risk group (being exposed in the same room or bathroom) compared to 0.6% and 0% in the medium and low risk groups, respectively (Table 2). There was a significant trend to higher rates of transmission by risk level of environmental exposure (*p* < 0.001). VRE transmission rate was 2.3% in the “staff” group (CoPat having the same medical care team), considerably lower than in the high risk environmental group (Table 2). No positive results were found when screening took place more than eight weeks after the exposure. The colonized patients had a median age of 80 (IQR 67–86) years and were male in 44.4%. Colonization was detected with the first screening in 85.2% and in second screening in 14.8%. No new colonization was detected in the third screening. Screening of patients at risk could not be completed in 7.7% of the patients at risk due to VRE-non-related death and in 5.8% of the patients due to loss of follow-up.

**Table 2:** VRE-positive patients and contact patients (CoPat) stratified by VRE *vanA/B* and risk type

	Overall	High risk^1^	Medium risk^2^	Low risk^3^	“staff”^4^	*p* value*
*VRE vanA/vanB*						
Number of patients at risk, N	1096	280	342	430	44	< 0.001
VRE-positive patients, N (%)	25 (2.3)	22 (7.9)	2 (0.6)	0 (0)	1 (2.3)	
*VRE vanA ST375*						
Number of patients at risk, N	681	120	295	262	4	< 0.001
VRE-positive patients, N (%)	8 (1.2)	6 (5.0)	2 (0.7)	0 (0)	0 (0.0)	
*VRE vanB ST117*						
Number of patients at risk, N	486	169	89	188	40	< 0.001
VRE-positive patients, N (%)	17 (3.5)	16 (9.5)	0 (0)	0 (0)	1 (2.5)	

Some patients were exposed to both VRE *vanA* ST375 and *vanB* ST117 (see Fig. 1)

^1^High risk: sharing the same room or bathroom with a VRE-colonized patient

^2^Medium risk: hospitalization in the same room immediately after a VRE-colonized patient’s discharge until terminal disinfection including UVc disinfection

^3^Low risk: sharing the same room within 3 weeks before the VRE-colonized patient was hospitalized in index room

^4^Same medical care team: all patients screened because of clinical suspicion during the outbreak by having the same medical care team as the index patient and therefore being screened with ward-wide screenings

*Cochran-Armitage test for linear trends

## Discussion

After experiencing one outbreak with *E. faecium vanA* ST 375 and two outbreaks with *vanB* ST117 from 2018 to 2019, we developed a new risk stratification for CoPat exposed to VRE-colonized or—infected patients. This risk stratification may allow to improve the yield of VRE-screening based on the type of exposure to a VRE-positive patient in a very low endemic setting. According to our results, low-risk and medium-risk patients do not necessarily need immediate screening or even preemptive contact isolation. It allows more rapid screening of patients with high exposure risks and same medical care team patients (“staff” group) saving time and financial resources. Our findings show that our proposal of risk-based screenings improves the yield of screenings of CoPat. In the literature, definitions of high-risk CoPat exposed to VRE are often ambiguous and ill defined. Known risk factors for VRE acquisition in an outbreak setting are hospitalization on the same ward as an index patient without contact precautions, antibiotic treatment with one or more antibiotics, alcoholism, and dementia [12]. Our study adds a risk stratification of exposed patients by type of exposure to an index patient depending on location at time of hospitalization as well as exposure to the same medical care team. Since no positive results occurred in the screenings performed after eight weeks, further stratification of the high-risk patients by time after exposure should be evaluated. This observation is supported by the results of Sohn et al. describing a median colonization time of 5.57 weeks [20]. The “staff” group should be analyzed in further studies taking account of additional risk groups. However, our study adds to the knowledge of screening that one can safely target at high-risk group, and keep medium- and low risk groups as option, if an outbreak continues despite contact precautions of the identified patients by high-risk and “staff” group screening.

Our study has three important limitations. First, the study was conducted in a low endemic setting and is not necessarily applicable in high endemic settings. However, it allowed us to better define such risk groups and there are still many low endemic countries for VRE. In addition, our results might also be useful for high endemic regions. Second, even though we experienced outbreaks with two different types of VRE, other strains might have different outbreak potential and risk stratification might be different. Third, the study is based on clinical data collection based on strictly followed standard operation procedures during outbreaks, but data are generated from a single center, and from a observational study. However, such clinical studies reflect daily practice in hospitals, and therefore, might be more generalizable than from randomized controlled trials with well standardized protocols.

## Conclusion

In conclusion, we propose a new risk stratification for screening of CoPat exposed to VRE during in-hospital outbreaks in a low endemic setting stratified by exposure during hospitalization. A focus on screening of high-risk exposed patients and patients taken care of by the same medical care team appears to be the most effective approach to detect transmissions for VRE. This new proposal requires further testing to adjust and improve such a stratification in other settings.

## Data Availability

All read data has been submitted to the European Nucleotide Archive (ENA) under project number PRJEB41046.

## Abbreviations

cgMLST: Core genome multilocus sequence typing
CoPat: Contact patients
CPGN: Carbapenemase-producing Gramnegatives
*E. faecium*: *Enterococcus faecium*
ENA: European Nucleotide Archive
ESBL: Extended spectrum β-lactamase
MDRO: Multidrug-resistant microorganisms
MLST: Multilocus sequence typing
MRSA: Methicillin-resistant *Staphylococcus aureus*
UVc: Ultraviolet C
VRE: Vancomycin-resistant enterococci
WGS: Whole genome sequencing
